# Mitochondrial cardiomyopathy with skeletal muscle myopathy caused by m.3260A > G mutation in *MT-TL1* gene: a case report

**DOI:** 10.1186/s13256-025-05633-0

**Published:** 2025-11-06

**Authors:** Anda Kadiša, Ritvars Vereskuns, Mihails Tarasovs, Ieva Mičule, Inna Inashkina

**Affiliations:** 1 Department of Internal Medicine, Riga East University Hospital, Riga, 1079 Latvia; 2 Residency Department, Riga Stradiņš University, Riga, 1007 Latvia; 3 Clinic of Medical Genetics and Prenatal Diagnostics, Children’s Clinical University Hospital, Riga, 1004 Latvia; 4 Latvian Biomedical Research and Study Centre, Riga, 1067 Latvia

**Keywords:** Case report, Mitochondrial myopathy, Mitochondrial cardiomyopathy, m.3260A > G, Heteroplasmy

## Abstract

**Background:**

Mitochondrial myopathies are a group of rare hereditary disorders that primarily affect muscle tissue, and present with muscle weakness, fatigue, exercise intolerance, and muscle pain. A key aspect of mitochondrial myopathies is the involvement of different organ systems, such as the cardiovascular system. Various disease features, including clinical and genetic heterogeneity, pose serious difficulties in the diagnostic process. We report a case of a young adult male, who presented with findings that suggested myositis, and was diagnosed with skeletal muscle myopathy and mitochondrial cardiomyopathy, caused by a m.3260A > G variant in the *MT-TL1* gene.

**Case presentation:**

A 22-year-old Latvian Caucasian man presented with a half-year history of fatigue, weakness, heaviness in the chest, and loss of breath, as well as a swollen right lower leg for about a week. Laboratory findings revealed increased creatine phosphokinase, creatinine kinase MB, and troponin T levels. All performed autoantibody tests for autoimmune disorders were negative, and no evidence of paraneoplastic syndrome was found. During repeated stays in the hospital, the patient developed heart failure and experienced decreasing muscle strength and muscle pain throughout the whole body. Following an unsuccessful therapy with corticosteroids and later L-carnitine, addition of thiamine lead to an overall improvement in the patient’s condition. Based on the family history, a genetic test was performed, which revealed a m.3260A > G variant in the *MT-TL1* gene.

**Conclusion:**

Several factors cause the process of establishing the correct diagnosis of mitochondrial myopathies and cardiomyopathies to be challenging. The presented case aims to raise awareness of rare mitochondrial myopathies, to help clinicians speed up the diagnostic process.

**Supplementary Information:**

The online version contains supplementary material available at 10.1186/s13256-025-05633-0.

## Background

Myopathies are a group of either inherited or acquired disorders, primarily affecting muscle tissue, and causing progressive structural changes and functional impairment [1]. Mitochondrial myopathies are a clinically diverse group of inherited myopathic disorders, stemming from mutations in either mitochondrial DNA (mtDNA) or the nuclear DNA (nDNA). They can present at any age, and, usually, are a part of a syndrome involving multiple organ systems [2].

Mitochondrial disorders are a heterogeneous group of diseases, however they share key features in their pathophysiological mechanisms—they affect mitochondrial respiratory chain function, oxidative phosphorylation, and cellular energy production. Mitochondrial diseases are rare, occurring in approximately 1 out of 5000 births, and their impact, usually, is the most severe on organs with the highest energy demands, including skeletal muscle and heart [2, 3]. As such, myopathies are among the most common manifestations of adult-onset mitochondrial disorders [2].

Diagnosing mitochondrial disorders, and mitochondrial cardiomyopathies (MCM) in particular is difficult, owing to the clinical and genetic heterogeneity [2, 4]. They typically can present with muscle weakness, exercise intolerance, and premature fatigue, after only mildly physically challenging activities [5]. Some of the features that can raise suspicion of mitochondrial disorders include concurring involvement of several organs with high metabolic demand, without a clear common cause, as well as disease manifesting early in life (before the age of 40), with unusual symptoms, such as stroke, hearing loss, and diabetes, or a maternal family history of multisystem symptoms. [6, 7].

**Fig. 1:**
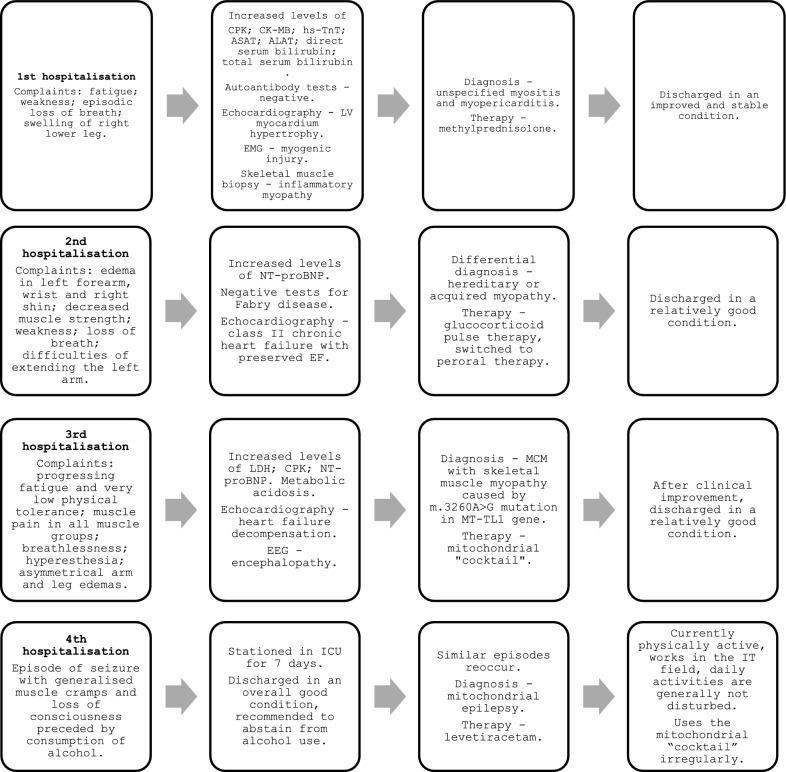
Chart mitochondrial myopathy R1. *ALAT* alanine aminotransferase, *ASAT* aspartate aminotransferase, *CK-MB* creatinine kinase MB, *CPK* creatine phosphokinase, *EEG* electroencephalogram, *EF* ejection fraction, *EMG* electromyography, *hs-TnT* high sensitivity troponin T, *ICU* intensive care unit, *LDH* lactate dehydrogenase, *LV* left ventricle, *MCM* mitochondrial cardiomyopathies

This paper presents a young adult male patient with uncertain history, symptoms, and clinical and laboratory findings, suggesting myositis, that led to diagnosis of skeletal muscle myopathy and MCM, caused by a m.3260A > G genetic variant in the *MT-TL1* gene. A chart summarizing key events can be seen in Fig. 1.

## Case report

A 22-year-old Latvian Caucasian man was transferred from a regional hospital where he presented with complaints of fatigue and weakness, that increase with physical activity, as well as feelings of heaviness in the chest and episodic loss of breath, that he had been experiencing for approximately half a year. In addition, he reported having a swollen right lower leg for about a week. Investigations performed at the regional hospital were notable with electrocardiogram (ECG) results that indicated pericarditis, as well as laboratory findings displaying raised creatine phosphokinase (CPK) and troponin T levels.

On arrival an ECG was performed, showing ST segment elevations, and PR segment depressions in leads V2–V6, without typical reciprocal changes, consistent with pericarditis. Physical examination revealed overall weakness, as well as weakness in distal and proximal muscle groups of upper and lower extremities. Notably, there were no signs of extraocular muscle weakness, such as ophthalmoplegia or ptosis. In addition, no remarkable vision impairment was observed. Laboratory studies were remarkable for increased levels of CPK (1755 U/L; reference range 39–190 U/L), creatinine kinase MB (CK-MB) (148 U/L; reference range 0–25 U/L), high sensitivity troponin T (hs-TnT) (86,24 ng/L; reference range 0–14 ng/L), aspartate aminotransferase (ASAT) (218 U/L; reference range 8–40 U/L), and alanine aminotransferase (ALAT) (67 U/L; reference range 8–41 U/L), direct serum bilirubin (9,3 mkmol/L; reference range 0–5 mkmol/L) and total serum bilirubin (33 mkmol/L; reference range 5–21 mkmol/L), as well as positive tests for urogenital chlamydiosis and ascariasis, for which an appropriate therapy was prescribed. Serological tests for other infections [including viral hepatitis and human immunodeficiency virus (HIV)] were negative. All performed autoantibody tests for autoimmune rheumatic disorders, including myositis-specific antibodies (anti-Jo-1), as well as myositis-associated antibodies (anti-U1-RNP, anti-Ro52, anti-SSB and anti-PM-Scl) were negative. Echocardiography displayed left ventricle (LV) myocardium hypertrophy, chest computed tomography—hepatomegaly. Chest x-ray and ultrasonography (USG), as well as heart magnetic resonance imaging (MRI) showed no considerable changes. Electromyography (EMG) results showed myogenic injury in all observed muscles, with higher prevalence in leg muscles, but no data of polyneuropathy in arm and leg nerves. There were no data indicating paraneoplastic syndromes. Skeletal muscle biopsy, obtained from the right shin, showed interstitial edema, light fibrosis, CD8 T-lymphocyte perivascular and intravascular infiltration—signs consistent with inflammatory myopathy, likely—polymyositis.

Based on the muscle weakness in both extremities, elevated CPK and histological findings in the muscle biopsy, a clinical diagnosis of unspecified myositis and myopericarditis was established. The patient received appropriate methylprednisolone therapy. During hospitalization the patient’s condition had improved, and he was discharged home in stable condition, with recommendations to continue the treatment, under the care of a family physician.

At 5 months after the first admission, the patient returned to the hospital with complaints of edema in the left forearm, wrist, and right shin, which was present almost every day, and worsened during the day, pain in muscles and decreased muscle strength throughout the whole body, difficulties climbing stairs and loss of breath during physical activities. The patient also remarked of difficulties of extending the left arm, after it has been contracted for a long time at the elbow joint. Following the initial improvements in muscle strength, due to glucocorticoid treatment, muscle weakness had gradually returned. Based on echocardiography findings, that showed symmetrical hypertrophy of the myocardium of the left ventricle as well as dynamic increase of NT-proBNP (from 337 pg/mL during the previous hospitalization, to 1172 pg/mL; reference range 0–125 pg/mL), class II chronic heart failure with preserved ejection fraction (EF) of 60% was discovered. In addition, hepatomegaly, small amounts of ascites and lymphostasis in the right leg were discovered. Again, no evidence of malignancy was found. Fabry disease was suspected; however, results of a blood test for the enzymatic activity of alpha-galactosidase turned out to be within normal range, thus not confirming this diagnosis.

The patient received glucocorticoid pulse therapy; however, subsequently, he started to complain about palpitations and worsening of shortness of breath. The pulse therapy was stopped and switched to oral methylprednisolone. After a gradual decrease of CPK (from 924 U/L at the beginning of the second hospitalization, to 542 U/L and 476 U/L), an increase of CPK level was observed (723 U/L). In addition, the lymphostasis in the right leg remained, and a light edema on the left wrist joint was observed. However, muscle weakness had slightly improved. History, gathered from the patient’s mother, revealed that already during his school years, the patient had avoided continuous physical activities.

At this moment, differential diagnosis between hereditary and acquired myopathy was considered. It was decided to continue treatment with methylprednisolone and perform another magnetic resonance imaging (MRI) examination of the heart, as well as Holter monitoring, both of which displayed no notable changes. It was decided to review the previous muscle biopsy specimen. The patient was discharged in a relatively good condition, with diagnosis of unspecified myositis with myocardial damage.

Less than 2 months later the patient returned to the hospital once again, with progressing fatigue and very low physical tolerance (the patient reported not being able to climb stairs to the third floor), as well as muscle pain in all muscle groups, particularly in the muscles of the back, breathlessness, hyperesthesia in arms and legs, as well as asymmetrical arm and leg edemas, more pronounced in the left arm and right leg. Blood tests revealed an overall increase in laboratory parameters, compared with the previous admission, specifically lactate dehydrogenase (LDH) (1706 U/L; reference range 135–225 U/L), CPK (836 U/L) and NT-proBNP (2738 pg/ml), as well as metabolic acidosis. In addition, the patient’s heart failure had decompensated (EF = 55%). Electroencephalogram (EEG) displayed signs of encephalopathy. Physical examination revealed severe muscle atrophy, as well as contractures in both elbows. Extremity Doppler ultrasonography was repeated, showing no signs of vasculitis or deep venous thrombosis (DVT). Likewise, abdominal and thyroid USG were unremarkable.

The patient received symptomatic therapy, as well as L-carnitine; however, it brought no improvements, and after 5 days the patient developed diarrhea, and L-carnitine treatment was discontinued. Instead, thiamine was added, which improved the patient’s overall condition, including increasing tolerance of physical activities.

Based on the clinical findings, as well as family history, that included information about the patient’s grandmother’s family, in which two out of seven sisters died suddenly at an early age (around 45 years old), a mitochondrial disease was suspected, and after obtaining a blood sample, genetic investigations were started with mtDNA full-length sequencing analysis according to a previously published protocol by Pelena *et al*. [8]. A trial of patient stabilization with a mitochondrial “cocktail” consisting of L-carnitine and thiamine was commenced and resulted in clinical improvement. The patient was discharged in a relatively good condition.

Subsequently, based on the results of the genetic testing, a diagnosis of MCM with skeletal muscle myopathy caused by a pathogenic variant in the *MT-TL1* gene (m.3260A > G transition) with a heteroplasmy level of 50% was established. Analysis of mtDNA from blood of the asymptomatic mother revealed 25% heteroplasmy of the same pathogenic variant.

After establishing mitochondrial dysfunction as the cause of the disease, the patient continued therapy with the aforementioned mitochondrial “cocktail” with the addition of coenzyme Q10 (CoQ10) and riboflavin.

Subsequently, muscle weakness and atrophy, as well as hypertrophic cardiomyopathy progressed. At the age of 23, the patient’s weight was only 49 kg.

At 4 months after the last hospitalization, the patient was rushed to hospital after an episode of seizure with generalized muscle cramps and loss of consciousness, and stationed in an intensive care unit (ICU) in a severe state. According to the patient’s mother, this episode was preceded by headache, nausea, and vomiting, and in the previous evening the patient had consumed around 100 mL of beer. After 7 days in the ICU, the patient had gradually improved and was discharged in an overall good condition, with recommendations of strict abstinence from alcohol use.

Similar episodes reoccurred at least seven more times, over the span of 6 years, up until the time of the submission of this article. The severity was variable, ranging between mild cramps with headaches, nausea, increased blood pressure and disorientation without loss of consciousness to bilateral tonic–clonic seizures and clinical death. They were provoked by consumption of alcohol, cannabis, or use of electronic cigarettes. However, in some episodes the provoking factor was not clearly determined. Each episode was resolved successfully, without long lasting consequences. The patient was diagnosed with mitochondrial epilepsy and prescribed levetiracetam (due to mitochondrial disorder, the use of valproic acid was contraindicated—the patient was appropriately warned) and advised to abstain from consuming any recreational psychotropic substances. In addition, he was advised to maintain an adequate sleep schedule (sleeping at least 7 hours) and to avoid operating a motorized vehicle for at least 1 year after each seizure episode. He was also diagnosed with stage one arterial hypertension and had experienced at least one recorded episode of paroxysmal atrial fibrillation (AF). Subsequently, he was prescribed ramipril and bisoprolol.

Currently, the patient is physically active, works in the information technology (IT) field and his daily activities are generally not disturbed. He continues to use the mitochondrial “cocktail” and other prescribed medications irregularly.

## Discussion

Mitochondrial diseases are rare genetic disorders that impair mitochondrial oxidative phosphorylation [2]. However, they are some of the most common inherited neurological disorders—in adults, the prevalence of disease, stemming from mutations in both nDNA and mtDNA, is estimated to be 1 in 4300, while the birth prevalence of diseases caused by mtDNA mutation is 1 in 5000 [3]. As with other mitochondrial disorders, patients suffering from primary mitochondrial myopathies (PMM) exhibit diverse clinical phenotypes, including differing symptoms, variable age of onset and severity of disease, stemming from a large number of causative mutations. These features, as well as the lack of a definitive diagnostic standard, pose serious difficulties to clinicians, in establishing the correct early diagnosis [4].

In the case presented in this paper, the process of establishing diagnosis of MCM with skeletal muscle myopathy, caused by a m.3260A > G mutation in *MT-TL* gene, was challenging. Owing to the rareness of the causative mutation, and, as such—unusual presentation of the symptoms, several diagnoses, including more common ones, such as idiopathic inflammatory myopathy, and more unusual ones, such as Fabry disease, were considered, before arriving at the correct diagnosis. This had led to the subsequent worsening of the patient’s state, owing to the lack of adequate treatment and lifestyle changes.

The *MT-TL1* gene (also known as the tRNALeu(UUR) gene) itself is responsible for encoding mitochondrial transfer RNA for leucin (tRNALeu) which adds leucine to the polypeptide chain of mtDNA-encoded subunits during translation [9].

As previously mentioned, mitochondrial myopathies can present with a diverse set of clinical features. Highly varied phenotypical presentations frequently lead to diagnostic dilemmas and are among the most serious challenges in the process of diagnosis and treatment of any mitochondrial disorder [2, 10]. Clinical features can include a wide range of manifestations, such as various ocular conditions [11], endocrinopathies [12], neurological disorders [13], gastrointestinal [14] symptoms, and others [10]. The skeletal muscle involvement itself is one of the most common features of mitochondrial diseases in general, with symptoms ranging from muscle weakness, exercise intolerance, and exercise induced myalgia, to muscle cramps and muscle wasting [2, 10, 15]. The myopathy usually is slowly progressive and proximal muscles are affected more often; however, it can spread to distal muscles of extremities, as well as muscles of the neck and face [2, 10]. Notably, the presentation of myopathy along with multisystem involvement without a clear origin can be a sign that points to a mitochondrial cause, along with unusual disease severity and observed maternal inheritance pattern [10].

As with the patient presented in this paper, frequent initial complaints include exercise intolerance and progressive muscle weakness, as well as fatigue and muscle pain [16, 17]. Fatigue in particular has been observed as the most common patient-reported symptom [18]. Notably, various frequently seen manifestations that point to a mitochondrial origin of the disease, such as diabetes, hearing loss, as well as ophthalmoplegia and other ocular symptoms [17, 19], were not observed in the patient discussed in this paper. Another frequent manifestation is epilepsy [20], which was observed only a few years after the diagnosis of mitochondrial disease was established.

Patients with mitochondrial myopathy can suffer from a recognized clinical syndrome, such as chronic progressive external ophthalmoplegia (CPEO), Leigh syndrome, mitochondrial encephalopathy and lactic acidosis with stroke-like episodes (MELAS), neuropathy, ataxia, retinitis pigmentosa (NARP), for example; however, often their illness does not fit into any single specific syndrome, and they present with an oligosymptomatic, or overlapping disease phenotype [19, 21]. According to a study performed by analyzing the North American Mitochondrial Disease Consortium (NAMDC) registry, out of 666 participants, the most frequent syndromes were Leigh syndrome (97 individuals), followed by MELAS (71 individuals), CPEO (55 individuals), and Leber hereditary optic neuropathy (LHON) (28 individuals). However, the most common diagnosis was multisystemic disorder (113 individuals), not fitting into any classical syndrome [22]. Adult-onset mitochondrial disease generally presents more subtly than pediatric disease. In addition, adulthood onset disease usually does not manifest as a typical clinical syndrome. These cases can either manifest in adulthood for the first time, or, similarly to the case described in this paper, be only recognized in adulthood after a history of nonspecific symptoms starting in childhood [10].

A particularly noteworthy mitochondrial disorder is LHON—a major cause of mitochondrial blindness, affecting approximately 1 in 30 000 individuals, particularly younger patients between the ages of 15 and 35 years. It usually presents with painless bilateral progressive vision loss that rapidly reaches the lowest point in 4–6 weeks. Long-term prognosis is poor—most patients face severe and permanent vision loss (visual acuity worse than 20/400) [11]. It is important to remark that our patient did not have any notable vision impairment that could be attributed to mitochondrial disease. 

Usual initial biochemical screening tests include CPK, LDH, lactate, blood glucose, hemoglobin A1c, thyroid, and liver function tests [23]. Elevated plasma lactate is frequently seen in various mitochondrial diseases, including mitochondrial myopathy; however, it can often be only slightly increased, or even normal, especially in adults [19]. However, standard biomarkers are not sufficient for establishing diagnosis, nor disease progression monitoring in adults [24].

If the diagnosis of mitochondrial myopathy is unclear, EMG can be useful to rule out conditions with similar clinical and laboratory findings; however, similarly to the case presented in this paper, often the findings show a nonspecific myopathic pattern, and frequently can be normal [4].

Skeletal muscle biopsy is one of the most important diagnostic tools, and can show histochemical alterations in the skeletal muscle, which indicates mitochondrial dysfunction; however, it can often be unremarkable. Characteristic findings include “ragged red fibers,” “ragged blue fibers,” and COX (cytochrome c oxidase)—negative fibers. Various possible nonspecific findings include internal nuclei, abnormal alterations in fiber size, neural atrophy, and accumulations of glycogen and/or lipids [16]. The timing of the biopsy may depend on various factors. If there is an acute destruction of muscle cells reflected by very high CPK levels, the mitochondrial deficiency findings can be obscured by the overall muscle fiber destruction, thus it may be more informative after the acute episode has subsided [25]. If a genetic disorder would have been suspected initially, our tactics also would prefer first obtaining the genetic results and to seek the morphological diagnosis only if the less invasive testing has come back negative.

Initial cardiovascular testing includes ECG and echocardiography [17]. Hypertrophic cardiomyopathy, dilated cardiomyopathy, and LV noncompaction are the three main manifestations of MCM [26]. It can affect not only myocardium, but also other structures of the heart, such as aortic valves, coronary arteries, cardiac conduction system, or pericardium [27], including the patient discussed in this paper. A common initial finding is progressive diastolic dysfunction and heart failure with preserved EF [7]. It often progresses to LV hypertrophy and systolic dysfunction. Even in the absence of LV hypertrophy, several ECG findings can support the diagnosis of mitochondrial disease, such as short PR interval, T wave abnormalities, and various degrees of atrioventricular block [17, 27]. In fact, in mitochondrial diseases with cardiovascular involvement, minor ECG abnormalities are the most common manifestations [17]. Cardiomyopathy is more common in mitochondrial diseases that present with skeletal muscle myopathy [28]. It is also less commonly seen in adult-onset mitochondrial diseases, whereas 20–40% of children with various mitochondrial diseases develop cardiomyopathy [26, 29]. In total, mtDNA-related cardiomyopathy is estimated to affect at least 1 in 10 000–15 000 of the general population [21].

A fundamental part of diagnosing mitochondrial diseases is genetic counseling, including molecular genetic testing, which can reveal pathogenic abnormalities in either nDNA or mtDNA. Mitochondrial mutations most commonly are inherited (approximately 75%), but can also occur *de novo* (approximately 25%) [27]. There are more than 250 different pathogenic mtDNA variants, and, in the case of primary mitochondrial myopathies (PMM), they have been discovered in all 37 mtDNA genes [16, 21]. As such, one of the main challenges in the molecular diagnosis of mitochondrial diseases is to distinguish rare or novel causative mutations from nonpathogenic gene polymorphisms. Mitochondrial respiratory chain disorders can be caused by mtDNA mutations in almost 20% of cases, and the rest are caused by mutations in nDNA [27, 30]. The most common cause of mtDNA alterations are point mutations—they are found in 1 of every 200 newborns; however, most individuals remain asymptomatic due to carrying a mutated gene with low heteroplasmy level [27, 31]. In contrast, large-scale mtDNA deletions are rare (1.5 in 100 000)—they most commonly arise as *de novo* mutations and carry a less than 10% risk of transmission [27].

The m.3260A > G transition in the *MT-TL1* gene is an infrequently occurring point mutation, that has been observed to cause maternal myopathy and cardiomyopathy [32, 33], in particular—hypertrophic and dilated cardiomyopathy [21, 32]. It is also related to MELAS syndrome [32, 34]. Hypertrophic cardiomyopathy is the most common form in all patterns of mitochondrial disease, occurring in up to 40% of patients [21, 26], including the patient described in this paper. The absence of mtDNA specific cardiovascular phenotype poses yet another diagnostics challenge to the clinicians. In addition, specific variants can lead to various phenotypes, while similar phenotypes can stem from multiple different mtDNA mutations [21]. However, while cardiac involvement in patients with mitochondrial respiratory chain diseases is common, and the presence of cardiomyopathy drastically decreases the overall survival rate [17, 29, 35], the incidence of severe cardiovascular morbidities is rare, and the prognosis is usually favorable [17].

In addition to myopathy and cardiomyopathy, our patient showed signs of encephalopathy and had eventually developed seizures, subsequently being diagnosed with mitochondrial epilepsy. To our knowledge, mitochondrial epilepsy, caused by m.3260A > G transition, has only been reported in two families with MELAS syndrome [34, 36].

Gathering a thorough family history is an essential part of diagnosis. It is helpful in directing the genetic tests, as well as discovering a potential disorder in other family members—whenever possible, maternal transmission of the disease should be recorded over the preceding three generations. However, notably, mitochondrial diseases have a substantial clinical heterogeneity within affected families, which can make it difficult to establish an accurate genotype/phenotype association and is an additional diagnostic challenge [19, 27]. This variety of symptoms and their severity can be explained by the phenomenon of heteroplasmy—the presence of mutated and wild-type mtDNA in the same individual [37]. In addition, different ratios of mutated and wild-type mtDNA can be observed between multiple affected family members, as well as between various tissues in the same organism [19]. The threshold to exhibit clinical symptoms usually varies between mutation loads of 60% and 90% depending on the specific mutation, with higher percentages leading to more severe phenotypes [32, 38, 39]. Similar findings could be observed in our case; however, the patient had a heteroplasmy level of only 50%, while his asymptomatic mother had a level of 25%. Variable heteroplasmy levels of the same mtDNA variant can even lead to phenotypic expression corresponding to different clinical syndromes—for example, the same mutation in *MT-ATP6* gene with a mutation load of 60–90% causes the less severe NARP, while a load of more than 90% causes the more clinically severe maternally inherited Leigh syndrome (MILS) [11].

Notably, the heteroplasmy level observed in our patient was low (50%); however, he displayed multiple serious clinical features, including myopathy and epilepsy. This could be explained by the fact that the mtDNA used in analysis was obtained from a blood sample. Analyzing samples from other tissues (for example, muscle, nervous) would most likely have yielded markedly higher levels [34].

Nowadays, the pharmacological treatment of mitochondrial disorders involves using various combinations of vitamins and cofactors that are vital for normal mitochondrial function. The goal of this therapy is to manage the associated symptoms, by promoting key enzymatic reactions, while reducing excess of free radicals and accumulated toxic acyl coenzyme A (CoA) molecules [40].

It is our common practice to start empirical trial of mitochondrial cofactors as soon as the mitochondrial disease is suspected, especially in acute cases. The components of the “cocktail” can be varied, but similarly to the case presented in this paper, we usually include supplements, such as carnitine and thiamine.

Carnitine is a cellular compound that transfers long-chain fatty acids across the mitochondrial inner membrane—a process that is vital for mitochondrial *β*-oxidation of fatty acids and the esterification of free fatty acids that would otherwise be sequestered by CoA. Its supplementation is used to restore free carnitine levels leading to the removal of accumulating toxic acyl compounds [40]. However, shortly after beginning the therapy, our patient suffered from diarrhea, which is a known side effect of carnitine [40], and, due to the lack of effectiveness, this treatment was temporary halted.

Thiamine is used to augment pyruvate dehydrogenase activity, thus increasing the catabolism of pyruvate to acyl CoA [41]. It has shown a good effect in lowering the blood lactate levels in our previous patients. Similarly to carnitine, and other components of the “cocktail” used to treat this patient, it is also readily available.

After establishing the diagnosis, two more compounds were added: CoQ10 and riboflavin. CoQ10 is a vital component in mitochondrial electron transport chain shuttling electrons from complexes I and II to complex III. Supplementing CoQ10 restores electron flow, thus improving the clinical manifestations of various mitochondrial diseases.

Riboflavin is a flavoprotein precursor, thus, functioning as an important building block in aforementioned complexes I and II. It is effective in treating various mitochondrial diseases, particularly those with complexes I and II deficiencies [40, 41].

## Conclusion

Mutations in the mtDNA are a rare cause of skeletal muscle myopathies and cardiomyopathies. Owing to factors, such as significant clinical and genetic heterogeneity, as well as a relatively small number of patients, the process of establishing correct diagnosis in these cases is challenging. The therapy usually includes a mitochondrial “cocktail,” consisting of vitamins and cofactors, as well as lifestyle changes, such as avoidance of recreational psychotropic substances and certain medications, including valproic acid. The presented case aims to raise awareness of rare mitochondrial myopathies, in order to help clinicians to recognize disease patterns and speed up the diagnostic process so as to begin the appropriate treatment sooner.

## Supplementary Information

Supplementary material 1.

## Data Availability

Data sharing is not applicable to this article as no datasets were generated or analyzed during the current study.

## Abbreviations

AF: Atrial fibrillation
ALAT: Alanine aminotransferase
ASAT: Aspartate aminotransferase
CK-MB: Creatinine kinase MB
CoA: Coenzyme A
CoQ10: Coenzyme Q10
COX: Cytochrome c oxidase
CPEO: Chronic progressive external ophthalmoplegia
CPK: Creatine phosphokinase
DVT: Deep venous thrombosis
ECG: Electrocardiogram
EEG: Electroencephalogram
EF: Ejection fraction
EMG: Electromyography
hs-TnT: High sensitivity troponin T
ICU: Intensive care unit
LDH: Lactate dehydrogenase
LHON: Leber hereditary optic neuropathy
LV: Left ventricle
MCM: Mitochondrial cardiomyopathies
MELAS: Mitochondrial encephalopathy and lactic acidosis with stroke-like episodes
MILS: Maternally inherited Leigh syndrome
MRI: Magnetic resonance imaging
mtDNA: Mitochondrial DNA
NARP: Neuropathy, Ataxia, Retinitis pigmentosa
nDNA: Nuclear DNA
NAMDC: North American Mitochondrial Disease Consortium
PMM: Primary mitochondrial myopathies
USG: Ultrasonography

## References

[1] Menon D, Alnajjar S, Katzberg H, Barnett C, Bril V. Demographic and clinical determinants of the quality of life in adults with inherited and acquired myopathies. Eur J Neurol. 2023;30:2518–24. 37159489 10.1111/ene.15854

[2] Mancuso M, McFarland R, Klopstock T, Hirano M, Artuch R, Bertini E, *et al*. International workshop: outcome measures and clinical trial readiness in primary mitochondrial myopathies in children and adults. Consensus recommendations.16–18 November 2016, Rome, Italy. Neuromuscul Disord. 2017;27:1126–37. 29074296 10.1016/j.nmd.2017.08.006 PMC6094160

[3] Gorman GS, Schaefer AM, Ng Y, Gomez N, Blakely EL, Alston CL, *et al*. Prevalence of nuclear and mitochondrial DNA mutations related to adult mitochondrial disease. Ann Neurol. 2015;77:753–9. 25652200 10.1002/ana.24362 PMC4737121

[4] Khan NA, Govindaraj P, Meena AK, Thangaraj K. Mitochondrial disorders: challenges in diagnosis and treatment. Indian J Med Res. 2015;141(1):13–26. 25857492 10.4103/0971-5916.154489 PMC4405934

[5] Berardo A, DiMauro S, Hirano M. A diagnostic algorithm for metabolic myopathies. Curr Neurol Neurosci Rep. 2010;10:118–26. 20425236 10.1007/s11910-010-0096-4 PMC2872126

[6] Gallego-Delgado M, Cobo-Marcos M, Bornstein B, Hernández-Laín A, Alonso-Pulpón L, Garcia-Pavia P. Mitochondrial cardiomyopathies associated with the m.3243A>G mutation in the *MT-TL1* gene: two sides of the same coin. Rev Esp Cardiol. 2015;68(2):153–5. 25440178 10.1016/j.rec.2014.09.007

[7] St-Pierre G, Steinberg C, Dubois M, Senechal M. What the cardiologist should know about mitochondrial cardiomyopathy? Can J Cardiol. 2019;35:221–4. 30760430 10.1016/j.cjca.2018.11.018

[8] Pelnena D, Burnyte B, Jankevics E, Lace B, Dagyte B, Grigalioniene K, *et al*. Complete mtDNA sequencing reveals mutations m.9185T>C and m.13513G>A in three patients with Leigh syndrome. Mitochondrial DNA A DNA Mapp Seq Anal. 2018;29(7):1115–20. 29228836 10.1080/24701394.2017.1413365

[9] Finsterer J. Genetic, pathogenetic, and phenotypic implications of the mitochondrial A3243G tRNALeu(UUR) mutation. Acta Neurol Scand. 2007;116:1–14. 17587249 10.1111/j.1600-0404.2007.00836.x

[10] Mattman A, Sirrs S, Mezei MM, Salvarinova-Zivkovic R, Alfadhel M, Lillquist Y. Mitochondrial disease clinical manifestations: an overview. BCMJ. 2011;53:183–7.

[11] Chen BS, Harvey JP, Gilhooley MJ, Jurkute N, Yu-Wai-Man P. Mitochondria and the eye—manifestations of mitochondrial diseases and their management. Eye. 2023;37:2416–25. 37185957 10.1038/s41433-023-02523-x PMC10397317

[12] Al-Gadi IS, Haas RH, Falk MJ, Goldstein A, McCormack SE. Endocrine disorders in primary mitochondrial disease. J Endocr Soc. 2018;2(4):361–73. 29594260 10.1210/js.2017-00434 PMC5865537

[13] Finsterer J. Mitochondrial neuropathy. Clin Neurol Neurosurg. 2005;107(3):181–6. 15823672 10.1016/j.clineuro.2004.07.001

[14] Finsterer J, Frank M. Gastrointestinal manifestations of mitochondrial disorders: a systematic review. Ther Adv Gastroenterol. 2017;10(1):142–54. 10.1177/1756283X16666806 PMC5330602 28286566

[15] Taivassalo T, Jensen TD, Kennaway N, DiMauro S, Vissing J, Haller RG. The spectrum of exercise tolerance in mitochondrial myopathies: a study of 40 patients. Brain. 2003;126(Pt 2):413–23. 12538407 10.1093/brain/awg028

[16] Ahmed ST, Craven L, Russell OM, Turnbull DM, Vincent AE. Diagnosis and treatment of mitochondrial myopathies. Neurotherapeutics. 2018;15(4):943–53. 30406383 10.1007/s13311-018-00674-4 PMC6277287

[17] Limongelli G, Tome-Esteban M, Dejthevaporn C, Rahman S, Hanna MG, Elliott PM. Prevalence and natural history of heart disease in adults with primary mitochondrial respiratory chain disease. Eur J Heart Fail. 2010;12(2):114–21. 20083621 10.1093/eurjhf/hfp186

[18] Gorman GS, Elson JL, Newman J, Payne B, MacFarland R, Newton LJ, Turnbull DM. Perceived fatigue is highly prevalent and debilitating in patients with mitochondrial disease. Neuromuscul Disord. 2015;25(7):563–6. 26031904 10.1016/j.nmd.2015.03.001 PMC4502433

[19] Keogh MJ, Chinnery PF. How to spot mitochondrial disease in adults. Clin Med. 2013;13(1):87–92. 10.7861/clinmedicine.13-1-87 PMC5873718 23472503

[20] Ticci C, Sicca F, Ardissone A, Bertini E, Carelli V, Diodato F, *et al*. Mitochondrial epilepsy: a cross-sectional nationwide Italian survey. Neurogenetics. 2020;21(2):87–96. 31900734 10.1007/s10048-019-00601-5

[21] Bates MG, Bourke JP, Giordano C, d’Amati G, Turnbull DM, Taylor RW. Cardiac involvement in mitochondrial DNA disease: clinical spectrum, diagnosis, and management. Eur Heart J. 2012;33(24):3023–33. 22936362 10.1093/eurheartj/ehs275 PMC3530901

[22] Barca E, Long Y, Cooley V, Schoenaker R, Emmanuele V, DiMauro S, *et al*. Mitochondrial diseases in North America: an analysis of the NAMDC registry. Neurol Genet. 2020;6(2): e402. 32337332 10.1212/NXG.0000000000000402 PMC7164977

[23] Parikh S, Goldstein A, Karaa A, Koenig MK, Anselm I, Brunel-Guitton C, *et al*. Patient care standards for primary mitochondrial disease: a consensus statement from the Mitochondrial Medicine Society. Genet Med. 2017. 10.1038/gim.2017.107. 28749475 10.1038/gim.2017.107 PMC7804217

[24] Jackson MJ, Schaefer JA, Johnson MA, Morris AA, Turnbull DM, Bindoff LA. Presentation, and clinical investigation of mitochondrial respiratory chain disease. A study of 51 patients. Brain. 1995;118(Pt 2):339–57. 7735877 10.1093/brain/118.2.339

[25] Walters J, Baborie A. Muscle biopsy: what and why and when? Pract Neurol. 2020;20:385–95. 32503899 10.1136/practneurol-2019-002465

[26] Scaglia F, Towbin JA, Craigen WJ, Belmont JW, O’Brian Smith E, Neish SR, *et al*. Clinical spectrum, morbidity, and mortality in 113 pediatric patients with mitochondrial disease. Pediatrics. 2004;114(4):925–31. 15466086 10.1542/peds.2004-0718

[27] Mazzaccara C, Mirra B, Barretta F, Caiazza M, Lombardo B, Scudiero O, *et al*. Molecular epidemiology of mitochondrial cardiomyopathy: a search among mitochondrial and nuclear genes. Int J Mol Sci. 2021;22(11): 5742. 34072184 10.3390/ijms22115742 PMC8197938

[28] Dimmock DP, Lawlor MW. Presentation and diagnostic evaluation of mitochondrial disease. Pediatr Clin North Am. 2017;64(1):161–71. 27894442 10.1016/j.pcl.2016.08.011 PMC5130109

[29] Imai-Okazaki A, Kishita Y, Kohda M, *et al*. Cardiomyopathy in children with mitochondrial disease: prognosis and genetic background. Int J Cardiol. 2019;279:115–21. 30642647 10.1016/j.ijcard.2019.01.017

[30] Smeitink J, van den Heuvel L, DiMauro S. The genetics and pathology of oxidative phosphorylation. Nat Rev Genet. 2001;2(5):342–52. 11331900 10.1038/35072063

[31] Elliott HR, Samuels DC, Eden JA, Relton CL, Chinnery PF. Pathogenic mitochondrial DNA mutations are common in the general population. Am J Hum Genet. 2008;83(2):254–60. 18674747 10.1016/j.ajhg.2008.07.004 PMC2495064

[32] Zeviani M, Gellera C, Antozzi C, Rimoldi D, Morandi L, Villani F, *et al*. Maternally inherited myopathy and cardiomyopathy: association with mutation in mitochondrial DNA tRNA(Leu)(UUR). Lancet. 1991;338(8760):143–7. 1677065 10.1016/0140-6736(91)90136-d

[33] Mariotti C, Tiranti V, Carrara F, Dallapiccola B, DiDonato S, Zeviani M. Defective respiratory capacity and mitochondrial protein synthesis in transformant cybrids harboring the tRNA(Leu(UUR)) mutation associated with maternally inherited myopathy and cardiomyopathy. J Clin Invest. 1994;93(3):1102–7. 8132749 10.1172/JCI117061 PMC294050

[34] Nishino I, Komatsu M, Kodama S, Horai S, Nonaka I, Goto Y. The 3260 mutation in mitochondrial DNA can cause mitochondrial myopathy, encephalopathy, lactic acidosis, and strokelike episodes (MELAS). Muscle Nerve. 1996;19(12):1603–4. 8941275 10.1002/(SICI)1097-4598(199612)19:12<1603::AID-MUS10>3.0.CO;2-S

[35] Imai-Okazaki A, Matsunaga A, Yatsuka Y, Nitta KR, Kishita Y, Suigura A, *et al*. Long-term prognosis and genetic background of cardiomyopathy in 223 pediatric mitochondrial disease patients. Int J Cardiol. 2021;341:48–55. 34298071 10.1016/j.ijcard.2021.06.042

[36] Connolly BS, Feigenbaum AS, Robinson BH, Dipchand AI, Simon DK, Tarnopolsky MA. MELAS syndrome, cardiomyopathy, rhabdomyolysis, and autism associated with the A3260G mitochondrial DNA mutation. Biochem Biophys Res Commun. 2010;402(2):443–7. 20965148 10.1016/j.bbrc.2010.10.060

[37] Holt IJ, Harding AE, Morgan-Hughes JA. Deletions of muscle mitochondrial DNA in patients with mitochondrial myopathies. Nature. 1988;331(6158):717–9. 2830540 10.1038/331717a0

[38] Miyabayashi S, Hanamizu H, Nakamura R, Endo H, Tada K. Defects of mitochondrial respiratory enzymes in cloned cells from MELAS fibroblasts. J Inherit Metab Dis. 1992;15(5):797–802. 1434520 10.1007/BF01800024

[39] Tatuch Y, Christodoulou J, Feigenbaum A, Clarke JT, Wherret J, Smith C, *et al*. Heteroplasmic mtDNA mutation (T––G) at 8993 can cause Leigh disease when the percentage of abnormal mtDNA is high. Am J Hum Genet. 1992;50(4):852–8. 1550128 PMC1682643

[40] Parikh S, Saneto R, Falk MJ, Anselm I, Cohen BH, Haas R, *et al*. A modern approach to the treatment of mitochondrial disease. Curr Treat Options Neurol. 2009;11:414–30. 19891905 10.1007/s11940-009-0046-0 PMC3561461

[41] El-Hattab AW, Zarante AM, Almannai M, Scaglia F. Therapies for mitochondrial diseases and current clinical trials. Mol Genet Metab. 2017;122(3):1–9. 28943110 10.1016/j.ymgme.2017.09.009 PMC5773113

