# Supplementary figures and images for: Mitochondrial cardiomyopathy with skeletal muscle myopathy caused by m.3260A > G mutation in MT-TL1 gene: a case report
Source: J Med Case Rep. 2025 Nov 6;19:573. doi: 10.1186/s13256-025-05633-0 (PMC12593855; doi:10.1186/s13256-025-05633-0)

## Flow Diagram — Case Reports following the CARE guidelines

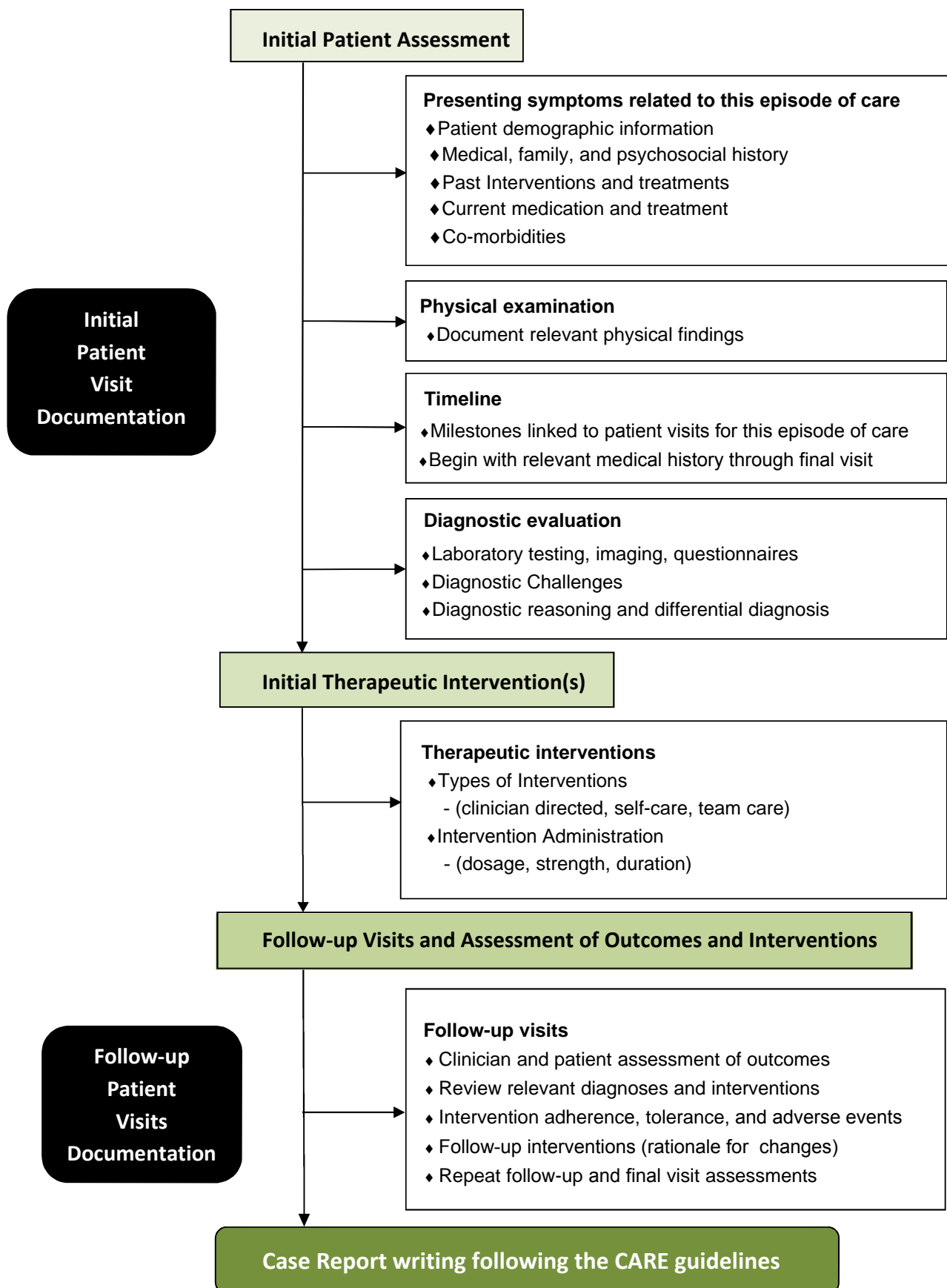

Supplement: Supplementary file 1 — Supplementary material 1. [file 13256_2025_5633_MOESM1_ESM.pdf]
